# Work-Life Balance vs. Work-Life Integration: A Young Physician Mother’s Journey

**DOI:** 10.1093/oncolo/oyac175

**Published:** 2022-09-06

**Authors:** Coral Olazagasti, Narjust Florez

**Affiliations:** Sylvester Comprehensive Cancer Center at the University of Miami, Miami, FL, USA; Division of Medical Oncology, Dana-Farber Cancer Institute, Harvard Medical School, Boston, MA, USA

## Abstract

This narrative describes the realities of balancing career and family for physicians in training.

*The authors present an account of the first author’s experience managing both a career in the medical field and responsibilities of a new mother. Written as a first-person narrative, it describes the joys and the challenges of the dual roles and offers advice to current and future mothers who are physicians in training.*

I aspired to be a doctor for most of my young adult life, and receiving my medical degree was one of my proudest moments. After that defining milestone, my journey became a series of stepping-stones comprised of professional goals that I intended to achieve. Early on I realized that having professional aspirations should not be at the expense of my personal life, and I naively believed that I could succeed as a doctor without sacrificing my own personal identities. I entered fellowship in hematology and oncology 2 months shy of my 30th birthday, and at that point, I was already married. The pressures of excelling in training, maintaining clinical responsibilities, developing research projects, and producing publications were enough to push the idea of motherhood to the back of my mind. Without realizing it, my career had taken a front-row seat in my life.

In the field of medicine, women often feel the need to delay family planning because of the stigma that having a family is equivalent to slower career development. Fortunately, I found many women who shared their own stories, much like my own, and in doing so highlighted the gender inequities for many women in academia. I realized I was the beneficiary of their outspoken words, and it was these women, whom I had never met in real life but admired from afar, who unknowingly empowered me to reclaim my personal life. I decided I could and should pursue my dream of becoming a mother and not let others make me believe I had to choose between the two. Then, on a summer day in 2020, we welcomed the love of my life, my son.

Motherhood quickly came with a reality check, ever more complicated by the COVID-19 pandemic and having family far away from us. I had envisioned experiencing the overwhelming sense of pure happiness and perfection that comes with a new baby. I did experience a heart full of happiness and unconditional love, but I also experienced periods of overwhelming and unexplainable sadness, often accompanied by bouts of uncontrollable crying. I could not understand my emotions in order to find a way to appropriately manage them. I was not entirely prepared for the roller coaster of feelings that came from a combination of sleep deprivation, the painful physical changes after childbirth, the uncertainties and mistrust of my ability to manage and become everything for a newborn life, the pressures of society to bounce back to your old self quickly and be “put together,” and most importantly, the terrifying reality that life is fragile. Instead of asking for help or advice, I put on a brave face and saved the crying for when I was alone. I was full of worries and anxieties, not sure if I’d be able to live up to my responsibilities successfully. Then there was also my professional career to think about.

Six days after bringing my son into this world and during my maternity leave, I scheduled my first interview for an academic faculty position. I was in no way mentally prepared to do so, but I feared that postponing my interviews would mean that I would lose opportunities and be passed on as a potential candidate. I surrendered to society’s and academia’s internal pressures. I felt compelled to do so in order to not “fall behind” and to stay at the same level as my peers, although my situation at the time was extremely different. During these interviews, I felt the need to hide my circumstances and decided to withhold sharing my new state of motherhood out of fear of being judged and it affecting others’ views of my ability to accomplish and excel in my professional goals. In academia, leadership frequently assumes that mothers, especially new mothers, are frail beings unable to carry on professional duties, and stigma exists that mothers are not emotionally and physically capable of managing both roles to full potential. Therefore, my new role of motherhood, one that was meant to be celebrated, felt like something I was better off omitting. The sad reality for me was that, to stay at the level of the playing field, I needed to downplay and even hide that I was a new mother.

Looking back at my 12 weeks of maternity leave, which I had meant to devote to the care of my newborn son, were spent trying to “have it all”, and I did my best to keep up with every aspect of my life. Besides caring for a newborn baby, I had managed to stay up to date on interviews, work emails, meetings, and several projects; I even attended conferences virtually. Despite all of this, I felt behind because I was not working as much as I “should” have. The unrealistic expectations I had for myself often did not allow me to give myself grace. Before I knew it, maternity leave was over and I was faced with the reality of returning to work, and with it the dread of placing my exclusively breast-fed son in the hands of a stranger.

There were minimal, if any, policies for supporting pregnant doctors, providing maternity leave, and assisting their return to work after giving birth, and there were even fewer national or organizational policies to help. I faced more challenges than I was prepared to encounter at the time.

I wish I could say I felt wholeheartedly supported, but that would not be true. I felt judged, and sometimes disrespected, particularly when it came to my needing to pump. It made me feel like I was failing as a physician, and in turn, I felt I was failing as a new mother. It was as if no one in the world understood how difficult adjusting to motherhood was, despite being surrounded by colleagues who were parents themselves. I was confused and still am, somewhat, today as to how doing something good for your child can be looked down upon in a professional setting, especially in the field of medicine. I felt ridiculed by people’s shocked reaction when I responded to the frequently asked question of “how long are you planning on breastfeeding?” by saying that my goal was to make it one full year. I lost count of the number of times I thought about quitting my breastfeeding relationship with my son because of the challenges I faced while pumping, which also inadvertently affected my supply. In efforts to accommodate the demands placed by others and attempt to successfully perform both roles to the best of my abilities, I purchased, out of pocket, the most expensive hands-off pump known to mankind. I figured that I could successfully wear the pump freely while not interrupting my clinical duties. And I did so during rounds, bone marrow aspirations, and biopsies, lunch, tumor boards, outpatient visits, etc. To me, this was my way of excelling and fulfilling all the work responsibilities during work hours while staying true to the goals I had set for my role as mother. In hindsight, however, shouldn’t institutions support a breastfeeding mother’s journey and allocate time and support for their needs? To me, this felt like one additional burden that fell on my shoulders, and I had to come up with the best solution. What a lonely and stressful journey it was, but a worthy one, nonetheless, because with determination and support from my spouse, I was able to reach my goal of one year of exclusively breastfeeding.

Today, what I have learned throughout this whole journey, is to give myself grace, to advocate for myself, feel pride in my accomplishments, and honor my commitments to the dual roles of a physician mom. I forgave myself for any mishaps and decided to live life day by day. Self-advocacy gave way to balance and, with it, passion in academic medicine. I get to pursue my passion of helping others, while always coming home every night feeling pure happiness and fulfillment when I walk into the door and get to cherish and enjoy my role as mama to the sweetest boy in the world. In hindsight, I wish I could have learned to juggle this new phase of life with the advice and support from others. I wish, as physician mothers, we did not have to feel as if openly discussing both roles would put us at a disadvantage to our male counterparts. I, personally, would go through every challenge again to be where I am today. Content, in every aspect of my life, and fulfilled as an oncologist and a mother. That much is true, and I am about to embark on the same journey for the second time. This time, I will apply the lessons I learned, which I also want to share with all physician mothers.

To current and future physician-in-training mamas, please remember:

1. You are worthy and valued.
2. Be your biggest cheerleader and advocate.
3. Give yourself grace, and do not feel shame in your dual roles.
4. Support others like yourself—share the happy and the challenging moments.
5. Love that baby! You are replaceable at work but never at home.

